# Population viscosity promotes altruism under density-dependent dispersal

**DOI:** 10.1098/rspb.2021.2668

**Published:** 2022-03-09

**Authors:** Jasmeen Kanwal, Andy Gardner

**Affiliations:** School of Biology, University of St Andrews, Greenside Place, St Andrews KY16 9TH, UK

**Keywords:** constant non-disperser principle, density-dependence, inclusive fitness, kin selection, limited dispersal, viscous population

## Abstract

A basic mechanism of kin selection is population viscosity, whereby individuals do not move far from their place of birth and hence tend to be surrounded by relatives. In such circumstances, even indiscriminate altruism among neighbours will often involve interactions between kin, which has a promoting effect on the evolution of altruism. This has the potential to explain altruistic behaviour across the whole tree of life, including in taxa for which recognition of kin is implausible. However, population viscosity may also intensify resource competition among kin, which has an inhibitory effect on altruism. Indeed, in the simplest scenario, in which individuals disperse with a fixed probability, these two effects have been shown to exactly cancel such that there is no net impact of viscosity on altruism. Here, we show that if individuals are able to disperse conditionally upon local density, they are favoured to do so, with more altruistic neighbourhoods exhibiting a higher rate of dispersal and concomitant relaxation of kin competition. Comparing across different populations or species, this leads to a negative correlation between overall levels of dispersal and altruism. We demonstrate both analytically and using individual-based simulations that population viscosity promotes the evolution of altruism under density-dependent dispersal.

## 1. Introduction

Kin selection is widely accepted as a key explanation for the evolution of altruistic behaviour [1–4]. One of the three basic mechanisms of kin selection is population viscosity, whereby individuals do not move far from their place of birth and therefore tend to be surrounded by relatives, such that even indiscriminate altruism will preferentially benefit kin [1,2,4]. This has the potential to explain altruistic behaviour across the whole tree of life, including in taxa for which recognition of kin is relatively less plausible, such as microorganisms [5].

However, while population viscosity tends to inflate relatedness between social partners, which acts to promote the evolution of altruism, it also tends to intensify kin competition for limiting resources, which acts to inhibit the evolution of altruism [2]. In the simplest scenario—of an infinite, inelastic island model in which every individual disperses independently and with a fixed probability—these relatedness and kin-competition effects exactly cancel such that there is no overall impact of rate of dispersal on the evolution of altruism [6]. This surprising result has stimulated a great deal of research attention, seeking to understand how different genetic, behavioural, and demographic factors can decouple the relatedness versus kin-competition effects of viscosity and hence modulate the evolution of altruism [7].

Here, we investigate how density-dependent dispersal modulates the evolution of altruism. We perform a mathematical analysis of an infinite, inelastic, island-model setting to show that the kin-competition consequences of altruism are reduced if individuals are more inclined to disperse when local population density is higher—which we term the ‘competition alleviation’ effect—and that individuals are favoured to employ such density-dependent dispersal—recovering Crespi & Taylor's ‘constant non-disperser’ principle [8]. The interplay of these two effects yields an overall negative relationship between the population-average rate of dispersal and the level of altruism, when comparing across different populations or species which vary with respect to the cost of dispersal. We derive explicit solutions for the evolutionary potential for altruism as a function of patch size and cost of dispersal, and we confirm these analytical results using individual-based simulations. Our analysis thereby demonstrates that population viscosity promotes the evolution of altruism under density-dependent dispersal.

## 2. Model and results

### (a) Mathematical model

Our model is based on Wright's classic ‘island model’ of population structure [9]. We assume an infinite population divided into discrete patches, with each patch containing *n* asexually breeding haploid individuals. In every generation, each breeder produces *k* offspring, and then dies. The offspring within each patch then interact socially in ways that influence their survival to adulthood. The probability that a focal juvenile survives is *S*(*y*, *y*′), where *y* is this juvenile's investment into altruism and *y*′ is the average investment into altruism made by the juveniles in her patch. We consider that altruism incurs a marginal survival cost to the actor, i.e. ∂*S*(*α,β*)*/∂α=−C<0*, and provides a marginal survival benefit to her patch mates, i.e. ∂*S*(*α*,*β*)/∂β=B>0. Surviving individuals may then attempt to disperse to randomly chosen, pre-existing (and already occupied) patches elsewhere in the population, with the focal individual's probability of attempting dispersal, x=M(P), dependent on the relative density of surviving individuals on her patch, $P=S(y^{\prime },y^{\prime })/S(\bar{y},\bar{y})$, where $\bar{y}$ is the population average level of altruism. A proportion *c* of individuals attempting dispersal do not successfully make it to another patch, but instead perish. Finally, *n* individuals in each patch are chosen at random to become the next generation of breeders, with all other individuals perishing, and this returns the population to the beginning of the life cycle. In this way, the population is inelastic, such that the number of breeding opportunities on a patch does not increase with the number of individuals who are competing for these breeding opportunities.

### (b) Potential for altruism

We analyse the above model using kin selection methodology [2,10,11], obtaining the condition for altruism to be favoured by natural selection as

2.1 $$\begin{array}{rl} & -C(1-c\bar{x})+B(1-c\bar{x})r-(1-\bar{x}-\mu )\frac{1-\bar{x}}{1-c\bar{x}}(B-C)r\\ & \;-\mu c(B-C)r>0, \end{array}$$

where $\bar{x}$ is the average rate of dispersal across the population, $\mu =\partial M/\partial P{|}_{P=1}$ is the dependency of dispersal on patch density, and *r* is the average relatedness of a focal individual to her patch mates (see electronic supplementary material for details of the derivation). This condition concerns the evolution of altruism across the entire population, rather than just for one particular patch. Expression (2.1) is a form of Hamilton's rule [1,2,12], and each of the four terms on the left-hand side of this condition readily yields an inclusive fitness interpretation. Specifically, an increase in altruism: (i) incurs a personal survival cost *C* for the focal individual, who would otherwise have survived the dispersal phase with probability $1-c\bar{x}$, (ii) provides a survival benefit *B* for patch mates, who go on to survive the dispersal phase with probability $1-c\bar{x}$, and who are valued by *r*, (iii) thereby gives a net boost *B* − *C* to the overall density of the patch, and these extra surviving individuals, who are valued by *r*, will fail to disperse away with probability $1-\bar{x}-\mu$, in which case they will compete for a breeding spot with other non-dispersing locals with probability $\left(1-\bar{x})/(1-c\bar{x}\right)$ of winning a breeding spot—in other words, this term captures the net change in kin competition due to increased patch density and increased dispersal, and (iv) leads to *μ* (*B* − *C*) extra dispersers, who suffer a mortality cost *c*, and are valued by *r*.

We can rearrange expression (2.1) into the form *C*/*B* < *A*, where *A* represents the threshold cost-to-benefit ratio above which altruism is not favoured and hence describes the ‘potential for altruism’ [13]. This is given by

2.2 $$A=\frac{r-ar-\chi r}{1-ar-\chi r},$$

where $a=[\left(1-\bar{x})/(1-c\bar{x}\right)]\times [\left(1-\bar{x}-\mu )/(1-c\bar{x}\right)]$ is the ‘scale of competition’ [11], i.e. the extent to which the marginal increase in patch density owing to altruism intensifies local competition for breeding opportunities, and $\chi =\mu c/(1-c\bar{x})$ represents the relative marginal increase in dispersal-related mortality owing to altruism. In the case of density-independent dispersal (μ=0, such that χ=0), expression (2.2) recovers the form A=(r−ar)/(1−ar) given previously by Gardner & West [14], which can be interpreted as the value of a focal individual's social partners measured relative to her average competitor.

The kin selection coefficient of relatedness is not a free parameter but is instead determined by the ecological and demographic context specified by the model, and can be expressed as

2.3 $$r=\frac{{\left(1-c\bar{x}\right)}^{2}}{{\left(1-\bar{x}\right)}^{2}+\bar{x}n(1-c)(2-(1+c)\bar{x})}$$

(see electronic supplementary material for derivation). From this, it follows that relatedness within the patch is a monotonically decreasing function of patch size (i.e. ∂*r*/∂n<0) and average dispersal rate $\left(\partial r/\partial \bar{x}<0\right)$, and is a monotonically increasing function of the cost of dispersal (∂r/∂c>0). Substituting these demographically explicit expressions for *a*, *χ* and *r* into expression (2.2) yields a demographically explicit form for the potential for altruism,

2.4 $$A=\frac{1}{n}+\frac{n-1}{n}\frac{(1-(1+c)\bar{x})\mu }{n\bar{x}(2-(1+c)\bar{x})+(1-(1+c)\bar{x})\mu }.$$

Accordingly, in general terms, we find that the potential for altruism is a monotonically increasing function of the density-dependence of dispersal (i.e. ∂*A*/∂μ>0), such that positive density-dependent dispersal has a promoting effect on altruism, on account of its competition-alleviating effect, and conversely, negative density-dependent dispersal has an inhibitory effect on altruism, on account of its competition-exacerbating effect.

### (c) Density-independent dispersal

We first consider the special case of density-independent dispersal (i.e. μ=0), whereby every individual disperses with the same probability, independently of the density experienced on their patch. In this scenario, expression (2.1) simplifies to

2.5 $$-C+B\;r-{\left(\frac{1-\bar{x}}{1-c\bar{x}}\right)}^{2}(B-C)\;r>\;0,$$

and expression (2.4) simplifies to

**Figure 1.**
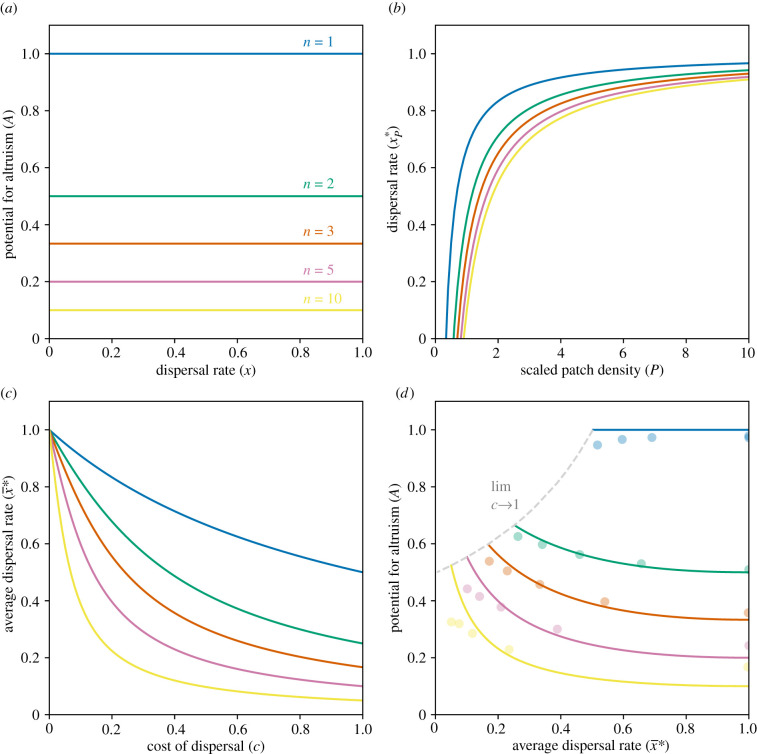
(*a*) The potential for altruism (*A*) is independent of the overall rate of dispersal (x) when dispersal is not conditioned upon local density (*μ* = 0), and is a monotonically decreasing function of patch size (*n*), as shown by Taylor [6] for costless dispersal (*c* = 0) and in the present analysis for costly dispersal (*c* > 0). (*b*) An individual's probability of dispersal $\left(x_{P}^{*}\right)$ is a monotonically increasing function of the relative density (*P*) of individuals in their patch, as shown by Crespi & Taylor [8] (here, the cost of dispersal is set to *c* = 0.5). (*c*) The overall rate of dispersal $\left({\bar{x}}^{*}\right)$ is a monotonically decreasing function of the cost of dispersal (*c*), as shown by Motro [15–17], Frank [18], and Taylor [19]. (*d*) The potential for altruism (*A*) is a monotonically decreasing function of the overall rate of dispersal $\left({\bar{x}}^{*}\right)$ when individuals are allowed to adjust their probability of dispersal according to local density (*P*), as revealed by the present analysis (solid lines represent analytical predictions and dots represent data from individual-based simulations). (Online version in colour.)

2.6 $$A=\frac{1}{n},$$

which recovers Taylor's key result that the level of altruism favoured by natural selection is independent of the rate of dispersal [6], and reveals that this also generalizes to scenarios involving non-zero cost of dispersal (i.e. c>0; figure 1*a*). In this special case of density-independent dispersal, the altruism-promoting effect of any increase in relatedness due to a reduction in the population-average rate of dispersal is directly cancelled by a corresponding altruism-inhibiting effect of an increase in the intensity of kin competition, such that altruism is no more favoured in a viscous population than it is in a well-mixed population.

### (d) Density-dependent dispersal

Next, we consider the scenario in which individuals are able to adjust their probability of dispersal according to the density experienced in their patch, and indeed that their dispersal strategy has been honed by natural selection such that they are behaving optimally in this respect. Upon the assumption of vanishingly low—but non-zero—variation in patch density across the population, we find that the optimal probability of dispersal for an individual experiencing relative patch density *P* is

2.7 $$\begin{array}{rl} x_{P}^{*} & =1-\frac{1}{P}\frac{c(1-c)}{r-c^{2}}\\ & =1-\frac{1}{P}\left(1-\frac{2}{1+2cn+\sqrt{1+4c^{2}n(n-1)}}\right) \end{array}$$

(if *r* > *c*; $x_{P}^{*}=0$ otherwise; see electronic supplementary material for derivation). Thus, we find that an individual's optimal probability of dispersal is a monotonically increasing function of the density of her natal patch (i.e. $\partial x_{P}^{*}/\partial P>0$; figure 1*b*) and, in particular, the probability that she does not disperse is inversely proportional to the density of her patch (i.e. $1-\;x_{P\;}^{*}\propto \;1/P$). This means that the absolute number of non-dispersing individuals within her patch $\left(P(1-\;x_{P\;}^{*})\right)$ is predicted to be completely independent of the patch density. In other words, all patches in the population are expected to retain the same number of non-dispersing individuals, with all of a patch's constituents in excess of this quota opting to disperse. This is the ‘constant non-disperser’ result of Crespi & Taylor [8].

The overall rate of dispersal across the population is then obtained by evaluating expression (2.7) at *P* = 1, which is the density of the average patch given our normalized definition of *P*. This yields

2.8 $${\bar{x}}^{*}=\frac{r-c}{r-c^{2}}\;=\frac{2}{1+2cn+\sqrt{1+4c^{2}n(n-1)}}$$

(if $r>c;\;{\bar{x}}^{*}=0$ otherwise). This exactly coincides with the optimal average dispersal rate for a population with no variation in patch density, as derived by Motro [15–17], Frank [18], and Taylor [19], and for the special case of n=1 it exactly recovers Hamilton's and May's [20] result (i.e. ${\bar{x}}^{*}=1/(1+c))$. From expression (2.8) we obtain that the overall rate of dispersal is a monotonically decreasing function of both patch size (i.e. $\partial {\bar{x}}^{*}/\partial n<0$) and the cost of dispersal $\left(\partial {\bar{x}}^{*}/\partial c<0\right)$—this is illustrated in figure 1*c*.

Having determined the optimal density-dependent dispersal strategy, we are now able to quantify the consequences for the evolution of altruism. Noting from expressions (2.7) and (2.8) that $\mu =\partial x_{P}^{*}/\partial P{|}_{P=1}=1\;-\;{\bar{x}}^{*}$, we find that this form of density-dependent dispersal leads to kin competition being completely abolished, as $a=[\left(1-\bar{x})/(1-c\bar{x}\right)]\;\times [\left(1-\bar{x}-\mu )/(1-c\bar{x}\right)]=0$ if $\mu =1\;-\;\bar{x}$. Accordingly, the kin-competition term (iii) in expression (2.1) vanishes altogether. Therefore, in this scenario, where dispersal rate is conditioned on patch density, any would-be increase in kin competition due to increased patch density is annulled by the corresponding increase in dispersal rate—any extra individuals whose survival is due to the increase in altruism disperse away rather than imposing a competitive strain on their relatives. In this case, increased kin competition no longer discounts the potency of viscosity as a mechanism for kin selection.

Making the substitution $\mu =1-\bar{x}=1-{\bar{x}}^{*}$ into expression (2.1) yields the resulting condition for natural selection to favour an increase in altruism

2.9 $$-C+B\;r-c^{2}(B-C)\;>\;0,$$

and making the same substitution into expression (2.4) yields the following expression for the potential for altruism

2.10 $$A=\frac{1-c{\bar{x}}^{*}}{1+((n-1)(2-(1+c){\bar{x}}^{*})-c){\bar{x}}^{*}}.$$

Mathematical analysis of this expression (see electronic supplementary material for details) reveals that the potential for altruism is a monotonically decreasing function of the number of breeders per patch (i.e. d*A*/d*n* < 0), with the direct impact of patch size on the potential for altruism being negative (∂A/∂n<0) and the indirect impact of patch size on the potential for altruism, via its impact on dispersal, being positive ($\partial A/\partial {\bar{x}}^{*}<0$ and $d{\bar{x}}^{*}/dn<0$, such that $\partial A/\partial {\bar{x}}^{*}\times d{\bar{x}}^{*}/dn>0$), and with the direct effect always outweighing the indirect effect (dA/dn = ∂A/∂n + $\;\partial A/\partial {\bar{x}}^{*}\times d{\bar{x}}^{*}/dn<0$). We also find that the potential for altruism is a monotonically increasing function of the cost of dispersal (d*A*/d*c* > 0), with its direct impact again being negative (∂A/∂c>0) and its indirect impact, via dispersal, again being positive ($\partial A/\partial {\bar{x}}^{*}<0$ and $d{\bar{x}}^{*}/dc<0$, such that $\partial A/\partial {\bar{x}}^{*}\times d{\bar{x}}^{*}/dc>0$), but with the indirect effect this time always outweighing the direct effect (i.e. dA/dc = ∂A/∂c  $+\;\partial A/\partial {\bar{x}}^{*}\times d{\bar{x}}^{*}/dc>0$). Crucially, we find that the potential for altruism is a monotonically decreasing function of the population-average dispersal rate (i.e. $\partial A/\partial {\bar{x}}^{*}<0$) when *n* > 1—in other words, limited dispersal results in an increased potential for altruism. This result is illustrated in figure 1*d*.

Rearranging expression (2.9) into the form *C*/*B* < *A*, we obtain an expression for the potential for altruism in terms of relatedness and the cost of dispersal. Further, by substituting in our expressions (2.3) and (2.8) for relatedness and the rate of dispersal, respectively, we also obtain an expression for the potential for altruism in terms of patch size and the cost of dispersal,

2.11 $$A=\frac{r-c^{2}}{1-c^{2}}=\frac{1}{n}\left(\frac{1-2c^{2}n+\sqrt{1+4c^{2}n(n-1)}}{2(1-c^{2})}\right).$$

If we compare expression (2.11), which describes the potential for altruism under the assumption that individuals optimally condition their dispersal behaviour to the density they experience on their patch, with the more general form given in expression (2.2), which describes the potential for altruism under arbitrarily density-dependent dispersal, then we can see how the kin-competition consequences of altruism have vanished here (*ar* = 0), and that the dispersal-induced mortality consequences of altruism are equal to the square of the cost of dispersal (*χr* = *c*^2^). More generally, we find that the right-hand side of expression (2.11) exceeds 1/*n* for all 0 < *c* < 1 and *n* > 1, such that the potential for altruism is higher in a viscous population than in a fully mixed population setting.

As an independent confirmation of this result, we have run a series of individual-based simulations (see electronic supplementary material for full details). In general, simulations provide a way of independently checking for errors in a mathematical analysis—if they recover the basic results, this lends further confidence to the analytical treatment. Moreover, they enable an assessment of the robustness of the results with respect to relaxation of simplifying assumptions made for the sake of analytical tractability (e.g. infinitely large populations, vanishingly low genetic variance and vanishingly weak selection). We simulated large populations (each consisting of 5 × 10^4^ breeders) with five different values of the cost of dispersal (*c* = 0, 0.2, 0.4, 0.6, and 0.8), for each of five different values of the number of breeders in each patch (*n* = 1, 2, 3, 5, and 10), to give a total of 25 separate simulations. The density-dependent dispersal rate (which, for simplicity, was approximated as a linear function of patch density in the simulations—as defined by its intercepts at two different values of patch density—thus reducing it to two independently evolving parameters) and level of altruism were free to evolve to their optimal values for each individual in the population. After 2 × 10^4^ generations, we recorded the population-average dispersal rate and determined the potential for altruism in each simulated population, and these data points are shown alongside the analytically predicted curves in figure 1*d*. Crucially, the simulation data demonstrate a negative relationship between the potential for altruism and rate of dispersal, in line with the prediction of our analytical model, and in contrast to Taylor's prediction [6]—shown in figure 1*a*—that the level of altruism is invariant with respect to dispersal. This negative relationship was found to be statistically significant using a Monte Carlo randomization test (*p* < 0.001 for 10^5^ random permutations of the data; see electronic supplementary material for details of this analysis).

## 3. Discussion

Taylor's analysis [6] revealed that the evolutionary potential for indiscriminate altruism is invariant with respect to dispersal rate under the simplest model of population structure, on account of an exact cancellation of the relatedness and kin-competition consequences of limited dispersal. This has thrown doubt upon the widely held view that population viscosity represents the most general mechanism by which kin selection drives the evolution of altruistic behaviour. Here, we have shown that Taylor's result crucially hinges upon his assumption that dispersal is density-independent: our analysis reveals that population viscosity promotes altruism under positive density-dependent dispersal and that it inhibits altruism under negative density-dependent dispersal. Moreover, we have shown that in the context of Taylor's model, if individuals are allowed to disperse conditionally upon local density, they are favoured to evolve a positive density-dependent dispersal strategy, and this leads to the complete elimination of the kin-competition consequences of increased altruism. In other words, simply by relaxing the assumption that every individual disperses with the same probability, and allowing individuals to optimize their dispersal behaviour conditionally on local density, we find that population viscosity emerges as a robust promoter of the evolution of indiscriminate altruism.

Under positive density-dependent dispersal, individuals are more likely to remain in sparsely populated patches and disperse away from densely populated patches. Such a strategy serves to reduce local competition for resources and smooth out variation in neighbourhood density across the wider population. Conversely, under negative density-dependent dispersal, individuals prefer to remain in patches with a higher density of individuals and disperse away from more sparsely populated patches. This strategy, wherein densely populated patches become even more dense, exacerbates local competition for resources. In investigating the effects of density-dependent dispersal on the evolution of altruism, we find that the potential for altruism is a monotonically increasing function of the density-dependence of dispersal—positive density-dependent dispersal promotes altruism, on account of its competition-alleviating effect, while negative density-dependent dispersal inhibits altruism, on account of its competition-exacerbating effect.

When we relax the assumption in Taylor's model [6] that all individuals disperse with the same probability, and instead allow them to condition dispersal rate according to the density of individuals in their patch, we find that a threshold-based positive density-dependent dispersal strategy evolves—if the number of offspring in a patch exceeds a population-wide threshold patch density, all offspring produced in excess of this threshold are favoured to disperse, with the remainder being favoured to remain in the patch. This is the ‘constant non-disperser principle’ described by Crespi & Taylor [8]. Subsequent models of the evolution of density-dependent dispersal in structured populations have similarly shown that positive density-dependent dispersal that maintains a population-wide threshold patch density is the optimal evolutionary strategy [21–27]. Insofar as variation in patch density is due to the level of altruistic behaviour occurring within the patch—i.e. an increased level of altruism is linked to a higher rate of offspring survival, and thus a higher patch density—then the constant non-disperser strategy leads to the complete elimination of the kin-competition consequences of altruism. Any extra individuals whose survival is due to an increase in altruism disperse away from the patch rather than imposing a competitive strain on their relatives. In this case, kin competition no longer discounts the potency of viscosity as a mechanism for kin selection, and thus population viscosity acts to promote the evolution of altruism.

Taylor's analysis [6] has motivated the development of an array of models which attempt to decouple the opposing effects of relatedness and kin competition by relaxing different assumptions made in his original model, and thereby shifting the relative scaling of competition and cooperation between kin. For example, population viscosity can be shown to promote altruism: when groups are able to expand in size [28] or into empty sites [29,30]; when traits may vary stochastically [31]; when individuals disperse in kin groups (i.e. ‘budding dispersal’), maintaining high within-group relatedness while reducing competition in the natal patch [14]; when there are sex differences in dispersal rate [13]; and when dispersal rates are allowed to differ between altruists and cheats, giving rise to a scenario wherein altruists disperse at a lower rate than cheats [32] (see Cooper *et al*. [33] for further examples). Other condition-dependent traits which may reduce kin competition include diapause or dormancy (i.e. a period of suspended or slowed development) and bivoltinism (the production of two generations of offspring per year, one of which develops and reproduces later than the other)—these processes can be framed as dispersal through time rather than space [34,35]. Additionally, in situations where one sex experiences more competition than the other, adjusting the sex ratio so as to invest more in the lower-competition sex may achieve similar results to altruistic dispersal [36]. Condition-dependent sex (switching reproductive mode from asexual to sexual when conditions become more challenging) might provide another means of reducing kin competition, by increasing the spread of offspring through ‘genotype space’ so that they occupy somewhat different niches [37]. Accordingly, density-dependent dispersal may represent just one illustration of a more general principle of competition-alleviation mechanisms acting to promote the evolution of altruism (see §1.2 of the electronic supplementary material).

In most of the extensions of Taylor's analysis, dispersal has been assumed to occur at a constant rate for all individuals in the population. Yet, empirical evidence suggests that condition-dependent dispersal is actually the norm among real-world organisms, with density being one of the main factors found to influence dispersal rate [38]. Indeed, density-dependent dispersal has been observed across a wide range of taxa, from wing-polymorphic insects including thrips [8], aphids [39], and parasitoid wasps [40], to fish [41], birds, and mammals [42], and even microorganisms, by means of quorum sensing [43–45]. To our knowledge, no model has previously considered how density-dependent dispersal impacts on the relationship between population viscosity and the evolution of altruism.

That population density is both *a determiner of* and *determined by* individuals' dispersal decisions makes density-dependent dispersal a particularly complex trait to model. Although our model predicts positive density-dependent dispersal as a mechanism for alleviating kin competition, we note that negative density-dependent dispersal is also commonly observed in nature. This has been attributed to the use of density as a proximate cue for habitat quality [38] or to the Allee effect [46]. We also note that in the empirical literature, dispersal is usually considered to consist of three stages—emigration, transiting, and immigration. However, in models of altruism in viscous populations which follow Taylor [6], only the factors involved in the decision to emigrate are considered in the treatment of dispersal—qualitative differences in the individuals who survive dispersal and settle in new patches, as well as environmental cues that may influence where an individual chooses to settle, are ignored. We acknowledge that there is scope for more realistic models with the inclusion of such considerations, but these are outside the scope of our model, which focuses on the consequences of relaxing one key assumption in the model of Taylor [6].

Taylor's analysis [6]—and much of the theoretical analysis that has emerged in its wake, including that of the present study—has considered an infinite island setting, as first introduced by Wright [9]. This simple model of population structure is one of the easiest to analyse, and thus remains a useful approach for assessing the broad-stroke evolutionary consequences of various demographic features [47]. Importantly, the result obtained by Taylor using the infinite island model approach—that any promoting effect of viscosity on altruism is directly cancelled by the corresponding inhibitory effect of increased kin competition—has been recovered in lattice-based models, in which social interactions and dispersal events occur only across spatially adjacent nodes [48,49], as well as in an abstract graph-theoretic setting that generalizes beyond these scenarios [50]. We therefore expect that our result will hold robustly across different population models, and extending the analysis to cover these represents an important avenue for future work. Another possible extension of our analysis would be to investigate how kin discrimination might modulate the evolution of altruism in this setting. Although kin discrimination may incentivize altruism toward social partners who are recognized as kin, it may also disincentivize altruism toward social partners who are not, and so it is unclear whether kin discrimination would promote, inhibit, or have no effect at all on the overall potential for altruism [51]. A particular complexity is that dispersal—itself an act of altruism—would be expected to depend on whether an individual's social partners are recognized as kin, such that kin discrimination might be expected to modulate the evolution of altruistic cooperation through multiple causal pathways.

Our model predicts an evolutionary relationship between density-dependent dispersal, competition alleviation, and altruism, for which clear, demonstrative empirical examples have yet to be identified. There is empirical evidence that density-dependent dispersal and avoidance of competition are directly linked (e.g. in black flies [52] and shore crabs [53]), and that density-dependent dispersal occurs in species that also display altruistic behaviours (such as aphids [54]). Plants are another domain in which density-dependent dispersal is extremely common, often involving dispersal polymorphisms, and there is growing evidence of altruistic behaviour in plants (see e.g. [55] and [56]). Comparative studies across such species may represent one avenue for seeking evidence of the three-way interaction predicted by our model. However, microorganisms may provide an ideal opportunity for experimental testing. In particular, in *in vitro* microorganism colonies that produce ‘public goods' chemicals, such as the pathogenic bacterium *Pseudomonas aeruginosa*, the level of altruistic behaviour is relatively easy to measure, and the medium can be easily altered to create high and low viscosity treatments. This paradigm therefore lends itself well to running tightly controlled experimental tests of the relationships between dispersal and altruism predicted by theory—see e.g. Kummerli *et al*. [57]. Previous experiments illustrate how dispersal patterns and scale of competition can be artificially manipulated by the experimenter to test their impact on the level of public goods production in the colony [58–60]. This paradigm might therefore be readily adjusted to contrast positive density-dependent, negative density-dependent, and density-independent dispersal, in both high and low viscosity media, to determine whether their predicted impact on the evolution of altruism is borne out.

## Data Availability

The code used to generate, analyse, and graphically visualize the simulation data is available at https://github.com/jkanwal/viscosity-promotes-altruism. The simulation data itself can be accessed at https://doi.org/10.17630/3d1808ce-e5ab-4d79-8ef5-bf30e26265e7 [61]. Further details of the mathematical analysis and individual-based simulations are provided in the electronic supplementary material [62].
